# Prevalence of Depression during Pregnancy in Spanish Women: Trajectory and Risk Factors in Each Trimester

**DOI:** 10.3390/ijerph18136789

**Published:** 2021-06-24

**Authors:** M. Carmen Míguez, M. Belén Vázquez

**Affiliations:** Department of Clinical Psychology and Psychobiology, Faculty of Psychology, University of Santiago de Compostela, 15782 Santiago de Compostela, Spain; mariabelen.vazquez.gomez1@rai.usc.es

**Keywords:** pregnancy, antenatal depression, prevalence, trajectory, risk factors

## Abstract

The aims of this research were to determine the trajectories of probable depression and major depression during pregnancy and to identify the associated and predictor variables (sociodemographic, pregnancy-related, and psychological) for both conditions in each trimester of pregnancy. A longitudinal study was carried out with 569 pregnant Spanish women who were assessed in the first, second, and third trimesters of pregnancy. Depression was assessed using the Edinburgh Postnatal Depression Scale and a clinical interview. Measures of anxiety and stress were also included. The prevalence of probable depression in the first, second, and third trimesters was 23.4%, 17.0%, and 21.4%, respectively, and that of major depression was 5.1%, 4.0%, and 4.7%. Thus, the prevalence of both conditions was the highest in the first and third trimesters. The trajectories of probable depression and major depression followed the same pattern throughout pregnancy. All of the psychological variables studied were associated with both conditions in all three trimesters, with perceived stress being a predictor at all times. The association between the other variables and both conditions of depression was similar. Two exceptions stand out: having had previous miscarriages, which was only associated with probable depression and was also a predictor, in the first trimester; and complications during pregnancy, which was only associated with probable and major depression in the third trimester. These findings should be taken into account in routine pregnancy follow-ups, and necessary interventions should be started in the first trimester.

## 1. Introduction

Depression is one of the most prevalent psychological disorders, affecting women at twice the rate of men [1]. In women, vulnerability to depression is particularly high during pregnancy and in the postpartum period [2], as the birth of a child is a life event associated with numerous biological, hormonal, psychological, familial, and social changes.

Antenatal depression has received less attention than postpartum depression [3], as pregnancy was traditionally thought to protect women against the onset or relapse of depressive disorders. Depression during pregnancy has important consequences, both for mothers and their children. In particular, the main consequence of antenatal depression for mothers is a continuation of the state into the postpartum period, as depression in pregnancy is the main risk factor for postpartum depression [4,5,6]. Children of mothers with antenatal depression have been found to be more likely to have intrauterine growth retardation [7], be born preterm [7,8,9,10], and be small for gestational age [9,10], all of which are major causes of neonatal and infant morbidity and mortality.

On the other hand, there is a lack of longitudinal research addressing the prevalence of antenatal depression in each trimester of pregnancy, as most studies are cross-sectional, which makes it impossible to determine the trajectory of depression throughout pregnancy. This can lead to an inaccurate picture of the actual situation, as the prevalence of depression often varies throughout pregnancy [11,12,13]. The analysis of the trajectory of depression in the same sample of pregnant women is important because it enables the identification of the times of the greatest vulnerability since cross-sectional studies provide very different results, as they use different assessment instruments.

The few existing longitudinal studies have provided mixed results regarding the trajectory of depression. In particular, some studies found that the first and third trimesters of pregnancy are the times of the highest prevalence of probable depression [13,14,15,16] and major depression [13], although the values in the third trimester did not reach those observed in the first trimester. However, in a study conducted in China [17], although probable depression was also the most prevalent in the first and third trimesters, the prevalence rate was higher in the latter than in the former. The same pattern was observed in Italy for major depression [18]. In all cases, the trajectory of depression is V-shaped. By contrast, a study in India [19] reported the opposite trajectory (/\), with the highest prevalence occurring in the second trimester and the lowest prevalence in the third trimester. On the other hand, a study conducted in Korea [20] found that the presence of probable depression followed an ascending path (/) between the first and the third trimester.

As longitudinal studies on the prevalence of antenatal depression by trimester are scarce, there is also a lack of knowledge regarding the associated variables at each point. Specifically, we did not find any studies in the existing literature that have analysed the variables associated with antenatal depression in each trimester. Thus, it is not possible to know whether there are variables associated with depression that remain stable throughout the pregnancy, or whether there are others that are specific to a particular trimester and then disappear in the rest of the pregnancy. Identifying such associations would enable the implementation of prevention and intervention measures adjusted to each phase. In this respect, the variables that are the most frequently associated with depression in pregnancy are sociodemographic, obstetric, and psychological variables [21]. In particular, antenatal depression has been associated with low educational level [15,22,23,24,25] and low socio-economic level [17,22,24,26,27,28,29], being unemployed [22,30,31], and not having planned the pregnancy [15,17,19,25,27,29,30,31,32,33,34]. Regarding psychological variables, having a history of depression [17,23,26,31], as well as anxiety [26,33,34,35,36] or stress [33,34,36] are the variables that are the most frequently associated with antenatal depression. On the other hand, in regard to variables such as age and parity, the studies’ findings are contradictory. Specifically, different studies have reported a relationship with younger age [18,37], while in others, it is related to older age [23,29]. Likewise, with respect to parity, both primiparity [36] and multiparity [19,23,26,31,32] have been associated with the presence of antenatal depression.

Monitoring women for depression throughout pregnancy will enable the identification of the most vulnerable phases and the variables associated with the presence of depression in each trimester. This would lead to more efficient help being offered in accordance with the real needs of future mothers and will prevent the depressive state and its associated consequences from extending to the postpartum period.

Therefore, the aims of the present research were first, to assess the trajectory of probable depression, assessed with a self-report instrument (EPDS), and of major depression, assessed with a clinical interview (SCID), during pregnancy; and second, to identify the associated and predictor variables (sociodemographic, pregnancy-related, and psychological) of both probable depression and major depression in each trimester of pregnancy. The study aimed to answer the following specific questions:

1. Is the trajectory of depression throughout pregnancy the same regardless of its severity?
2. Do the same variables predict the presence of probable depression and of major depression?
3. Which variables are the most closely associated with depression in each trimester? Are they the same, or are there trimester-specific predictors?

## 2. Materials and Methods

### 2.1. Procedure and Participants

**Figure 1:**
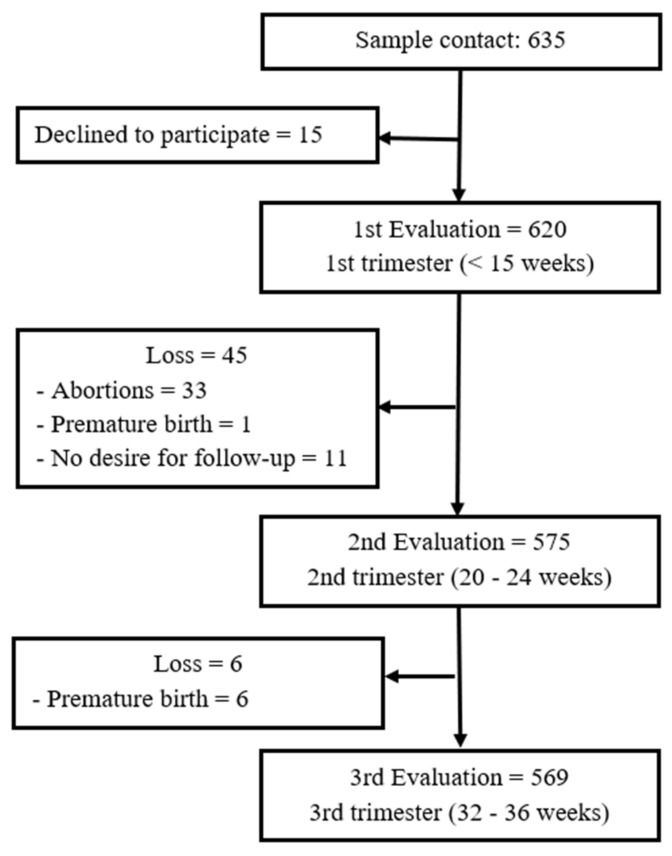
Flow diagram of recruitment and progress through the study.

The present research was conducted in accordance with the Helsinki Declaration and received previous approval from the ethics committees of all of the institutions involved. It was a longitudinal study with three assessment waves: the 1st trimester of pregnancy (M = 10.87 weeks; SD = 2.36), the 2nd trimester of pregnancy (M = 20.69 weeks; SD = 1.21) and the 3rd trimester of pregnancy (M = 33.28 weeks; SD = 2.05). Pregnant women attending the primary public healthcare service in northwest Spain were recruited in the 1st trimester of pregnancy (*n* = 620). Women were eligible to participate if they were at least 18 years of age, were in the first trimester of pregnancy, spoke Spanish, and were willing to participate in subsequent assessments throughout the pregnancy. The exclusion criteria were: being under 18 years of age, having a gestational age equal to or greater than 15 weeks, not reading or speaking Spanish, and not having participated in any of the evaluations. The aims and procedures were explained, and the pregnant women who were willing to participate provided written informed consent. The final sample consisted of 569 women. The procedure and sample tracking characteristics are shown in Figure 1.

All women participated voluntarily in the study. The assessments were carried out personally and individually at the hospital prior to the women entering the protocolised pregnancy follow-up consultation. In each pregnancy trimester, all of the questionnaires and SCID interviews were administered personally by one psychologist who received SCID training and who was blind to the EPDS scores. The average duration of each interview was about 40 min.

### 2.2. Measures

#### 2.2.1. Socio-Demographic and Pregnancy Information

Three ad hoc questionnaires including questions on socio-demographic (e.g., age, marital status, educational level, occupational status, and personal monthly income), pregnancy-related (e.g., parity, previous abortions, planned pregnancy, pregnancy reaction, complications, attendance at maternal classes), and psychological variables were elaborated on specifically for the study.

#### 2.2.2. Depression

The Edinburgh Postnatal Depression Scale (EPDS) [38] is a self-reported questionnaire consisting of 10 items with 4 response options. The scores range between 0 and 30, with higher scores indicating a greater severity of depression. The Spanish validation of the EPDS for use in pregnancy was used [13], which determined that the most appropriate cut-off point for screening for probable antenatal depression was ≥ 10. The reliability of the EPDS during pregnancy was 0.81 in the first trimester, 0.82 in the second trimester, and 0.85 in the third trimester in the present study.

The Structured Clinical Interview for DSM-IV (SCID) [39] is a semi-structured interview that determines a formal diagnosis according to the Diagnostic and Statistical Manual of Mental Disorders (DSM-IV). The use of such interviews improves diagnostic reliability by standardising the assessment process and increases the diagnostic validity by facilitating the application of DSM diagnostic criteria and the systematic enquiry of symptoms that might otherwise go unnoticed.

#### 2.2.3. Stress

The Spanish validation [40] of the Perceived Stress Scale (PSS) [41] was used. The PSS is a self-administered scale that measures the degree to which life situations in the previous month are rated as stressful. It consists of 14 items, with 5 response options. The scale ranges from 0 to 56, with higher scores indicating a higher level of perceived stress. With this sample, the reliability of the PSS during pregnancy was 0.86 in the first trimester, 0.88 in the second trimester, and 0.87 in the third trimester.

#### 2.2.4. Anxiety

The State-Trait Anxiety Inventory (STAI) [42] assesses both the current level of anxiety and the individual’s predisposition to suffering from anxiety. It consists of 40 items, 20 of which refer to the state subscale (STAI-E), with other 20 referring to the trait subscale (STAI-R). The score for each subscale ranges from 0 to 60, with higher scores indicating higher levels of anxiety. For the state subscale, the recommended cut-off point for women is greater than 31, and for trait anxiety, the cut-off point for woman is greater than 32 (75th percentile). In our sample, the reliability of the trait anxiety subscale in the first trimester of pregnancy was 0.88, and the reliability of the state subscale, 0.91. In the second and third trimesters, the reliability of the state anxiety subscale was 0.92.

### 2.3. Data Analysis

Data were analyzed using SPSS Statistics, version 22 (PASW Statistics for Windows, SPSS Inc., Chicago, IL, USA), and a significance level of *p* < 0.05 was applied. To test the differences between the presence or absence of depression, a chi-square test for discrete variables and Student’s t-tests for continuous variables were performed. Cramers’s V coefficients and Cohen’s d were calculated in order to estimate the size of the effect.

Binary forward stepwise logistic regression analysis was also used to determine the variables predicting depression in pregnancy, according to the EPDS, and/or major depression, according to the SCID, in each trimester of pregnancy. The dependent variable was probable depression status (EPDS ≥ 10, yes/EPDS < 10, no) or major depression status (yes depression/no depression), and the independent variables were those variables for which significant differences were found in the two-by-two analyses. Likewise, Cronbach’s alpha (α) was calculated in order to estimate the reliability of the scales.

## 3. Results

### 3.1. Characteristics of the Sample

The study sample was composed of 569 women ranging in age from 18 to 45 years, with a mean age of 32.80 years (SD = 4.75). Most of the women were married or cohabiting (94.9%); 46.4% (*n* = 264) had university education, and 35.3% (*n* = 201) had secondary education. Regarding employment status, 75.2% (*n* = 428) were working. Regarding personal monthly income, 44.6% (*n* = 254) stated that they earned less than 1000 euros per month. Pregnancy was planned in 85.9% (*n* = 489) of cases, 59.4% (*n* = 338) of the women were primiparous, and 93.0% (*n* = 529) reported having reacted positively to confirmation of the pregnancy. Likewise, 63.3% (*n* = 360) reported attending maternal education classes, with the mean attendance being 4.45 classes (SD = 4.33) out of the 10 classes offered on a regular basis.

### 3.2. Trajectory of Depression throughout Pregnancy

**Figure 2:**
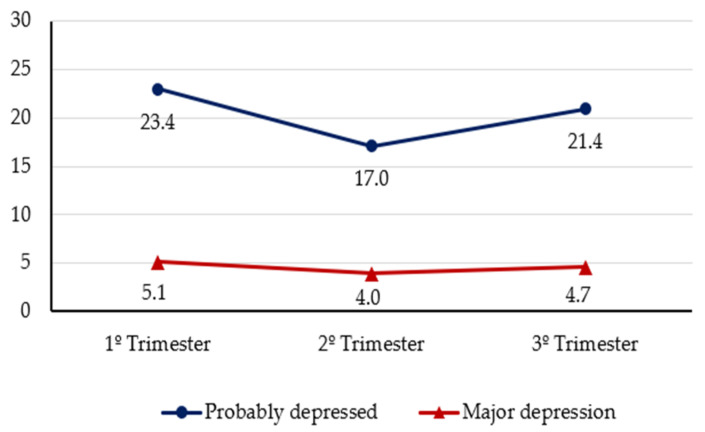
Trajectory of the prevalence of probable depression and major depression during pregnancy.

The prevalence of depression varied according to the time of the assessment and the assessment instrument used (Figure 2). The first trimester was the period during which the highest percentage of women had probable depression (23.4%) or major depression (5.1%). The prevalence of both probable depression (17.0%) and major depression (4.0%) was the lowest in the second trimester. In the third trimester, the prevalence of both probable depression (21.4%) and major depression (4.7%) was higher than in the second trimester but did not reach the values observed in the first trimester. Thus, the trajectories of probable depression and major depression followed the same pattern throughout pregnancy.

### 3.3. Variables Associated with the Presence of Depression

#### 3.3.1. Sociodemographic Variables

Regarding the sociodemographic variables, the characteristics of the pregnant women experiencing probable depression or major depression in each trimester of pregnancy as well as the variables associated with both types of depression are shown in Table 1. Regarding the mean age, significant differences were found in the prevalence of probable depression in the first (31.99 vs. 33.05; t(569) = 2.254, *p* = 0.025, d Cohen = 0.25) and second trimesters (31.63 vs. 33.04; t(569) = 2.686, *p* = 0.007, d Cohen = 0.28), as these women were younger than the women who did not experience probable depression. For women experiencing major depression, the mean age was statistically significantly lower in the second (t(569) = 4.158, *p* < 0.001, d Cohen = 0.82; 28.83 vs. 32.97) and third trimesters (t(569) = 4.768, *p* < 0.001, d Cohen = 0.86; 28.63 vs. 33.01).

Having a lower level of education was associated with both depression conditions in all three trimesters of pregnancy, while a lower level of income was only associated with probable depression in the 1st and 2nd trimesters. On the other hand, not having a partner, not cohabiting, or not being married was not associated with either condition at any time.

#### 3.3.2. Pregnancy-Related Variables

In terms of pregnancy-related variables, the characteristics of pregnant women with probable depression and major depression in each trimester of pregnancy as well as the variables associated with both conditions are shown in Table 2.

Having reacted negatively to the confirmation of pregnancy was associated with both probable and major depression at all time points. On the other hand, having had previous miscarriages was only associated with probable depression in the first trimester, while having had complications at some time during pregnancy was only associated with the presence of major depression in the third trimester.

#### 3.3.3. Psychological Variables

Regarding the psychological variables, the characteristics of pregnant women with probable depression and major depression in each trimester of pregnancy as well as the variables associated with both types of depression are shown in Table 3.

Regarding perceived stress, significantly higher mean scores were found among women experiencing probable depression and women experiencing major depression. Specifically, women with probable depression had higher mean scores for perceived stress in the first (t(569) = −14.332, *p* <0.001, d Cohen = −1.63; 25.44 vs. 16.17), second (t(569) = −15.11, *p* < 0.001, d Cohen = −1.81; 25.69 vs. 14.55) and third trimesters (t(569) = −14.946, *p* < 0.001, d Cohen = −1.59; 25.33 vs. 15.08). In addition, the mean scores were significantly higher among women with major depression in the first (t(569) = −6.234, *p* < 0.001, d Cohen = −1.30; 26.66 vs. 17.89), second (t(569) = −7.953, *p* < 0.001, d Cohen = −1.82; 28.52 vs. 15.94) and third trimesters (t(569) = −8.343, *p* < 0.001, d Cohen = −1.74; 29.00 vs. 16.69).

All of the psychological variables studied were associated with both probable and major depression at all times. Thus, having a prior history of depression, having experienced a worsening of mood in previous pregnancies, having elevated state and trait anxiety as well as a higher level of stress were significantly associated with both probable depression and major depression.

### 3.4. Predictors of Depression in Each Trimester

Predictors of the presence of probable depression and major depression in each of the trimesters are shown in Table 4 and Table 5.

Age, previous miscarriages, state anxiety, and perceived stress were predictors of probable depression in the first trimester. Specifically, being aged 30 years old or younger (OR = 2.55), having had previous miscarriages (OR = 3.28), having a high state of anxiety (OR = 3.97) as well as higher perceived stress (OR = 1.24) increased the likelihood of probable depression in the first trimester.

In the second trimester, having had probable depression in the first trimester (OR = 13.61) as well as higher perceived stress in the first (OR = 1.14) and second trimesters (OR = 1.35) increased the likelihood of having probable depression.

In the third trimester, the predictors of probable depression were having had major depression in the first trimester (OR = 5.43), probable depression in the second trimester (OR = 6.19), and an elevated state of anxiety (OR = 4.88) and higher perceived stress (OR = 1.16) in the third trimester.

In the first trimester, the predictors of major depression were having perceived that pregnancy had a negative influence on employment (OR = 3.20) and having higher perceived stress (OR = 1.13). In the second trimester, the predictors were being aged 30 years old or younger (OR = 1.26) and experiencing higher perceived stress (OR = 1.27). In the third trimester, the predictors were being aged 30 years old or younger (OR = 7.23) and experiencing higher perceived stress in the second (OR = 1.16) and in the third trimesters (OR = 1.18).

## 4. Discussion

### 4.1. Trajectory of Depression during Pregnancy

The aim of the present longitudinal study was to analyse the trajectory of both probable depression and major depression during pregnancy. The prevalence of probable depression ranged from 17.0% to 23.4%, and the prevalence of major depression ranged from 4.0% to 5.1%. During pregnancy, the variation in probable depression and major depression followed the same pattern or trajectory. In both cases, the prevalence was the highest in the first trimester, decreased in the second trimester, and increased again in the third trimester, although not reaching the rates observed in the first trimester. It is possible that, as stated by Rallis et al. [14], this trajectory of depression may occur because the beginning of a pregnancy can be a time of strong psychological vulnerability involving multiple factors, such as hormonal, physical, psychological, and emotional adjustment to the new situation of pregnancy, which can increase vulnerability to the development of depressive symptoms. On the other hand, the third trimester is another critical time, as it also involves major physical and emotional changes in view of the approaching birth.

The findings of the few studies that have assessed the presence of depressive symptomatology in all three trimesters of pregnancy are variable. The same pattern as the one observed in the present study was also observed in studies conducted in China [16], Turkey [15], and Australia [14]. However, in the study by Weng et al. [17] conducted in China, although the two most prevalent times of depressive symptomatology were also the first and third trimester, the prevalence was higher in the latter than in the former. On the other hand, the prevalence observed in a study in Korea [20] follows a rising pattern from the first to the third trimester, and Ajinkya et al. [19] found to have the highest prevalence of depressive symptomatology in the second trimester and the lowest values in the third trimester. However, it should be noted that these studies use different scales and cut-off points to assess probable depression, such as the EPDS [17], the BDI [15,19], and the SDS [16]. On the other hand, some studies have identified different latent trajectory groups based on the total scores of depressive symptoms [43,44].

Regarding the trajectory of major depression, we can only compare our data with those reported by Marchesi et al. [18] in Italy, as this is the only study including a longitudinal follow-up in the three trimesters of gestation. The trajectory reported is similar to that observed in the present study, following a V-shaped pattern, with the prevalence of depression being the highest in the first and third trimesters, although in this case, the prevalence was the highest in the third trimester.

The differences between the trajectory observed in the present study and those observed in other studies may be due to cultural differences, the quality of prenatal care received and the levels of satisfaction with this prenatal care, the professionals carrying out prenatal care (midwives and/or obstetricians), health conditions, and the type and accessibility of healthcare (public/private) in each country.

### 4.2. Variables Associated with Probable Depression and Major Depression in Pregnancy

The second objective of this study was to identify the sociodemographic, pregnancy-related, and psychological variables associated with both probable depression and major depression in each trimester. This will enable us to determine whether any variables are specifically associated with a particular trimester of pregnancy, with one condition of depression or the other, or with both equally.

Regarding the sociodemographic variables, both probable depression and major depression were associated with younger age. In addition, an age less than or equal to 30 years old was found to be a predictor of major depression in the second and third trimester. This relationship was also found in other research in which being younger than 25 [31], younger than 20 [22], or aged 15–20 [25] was associated with an increased risk of antenatal depression. This relationship can be explained by the fact that younger women tend to have a more unfavourable and unstable economic position and lower-paid jobs, leading to lower income level [25].

On the other hand, in all trimesters of pregnancy, both probable depression and major depression were associated with lower educational level. These findings are consistent with those of previous studies [15,22,23,25]. This relationship can be explained by the fact that a low level of education is often related to socio-economic disadvantage [23], which is one of the most worrying aspects for women when facing motherhood, as they may fear that they will not be able to meet their children’s needs [45]. Low socio-economic status is also often accompanied by increased stress, which is, in turn, considered a risk factor for depression. In the present study, lower income was associated with the presence of probable depression in the first and second trimesters. This finding is consistent with the findings of other authors, who observed associations with low socioeconomic status [22,24] and family income below the minimum wage [27]. Employment status is one of the factors related to both educational and economic status. Being unemployed was associated with the presence of probable depression in the first trimester and with major depression in the second and third trimesters, as was also found in other studies [22,30,31]. Giardinelli et al. [30] suggested that this relationship may be due to the fact that not working implies having a smaller social support network and some degree of isolation. Likewise, not being in paid work is associated with lower educational attainment and lower economic resources. Another possible factor explaining this association is the frustration that the women had after observing that being pregnant prevented them from having equal access to the labour market, as some women reported being dismissed from work, not being able to apply for positions of responsibility, or being obliged to request reduced working hours or job adaptations that often did not correspond to their professional category. This hypothesis would explain the observed association between the perception of the women that pregnancy had had a negative influence on their work situation and the presence of probable depression at all stages of pregnancy and with major depression in the first and second trimesters. It was also a predictor of major depression in the first trimester. Of note is a sociodemographic variable with which no association was found at any time during pregnancy with either probable or major depression. Not having a partner has been associated in some studies with antenatal depression [17,23,29], but not in this research. However, only 29 women claimed to not have a partner in the present study, and this could influence the results. On the other hand, the explanation for this finding could be that what is associated with depression is not so much with having or not having a partner, but with the quality of the relationship and how it is perceived by the woman as satisfactory or unsatisfactory.

Regarding pregnancy-related variables, parity, specifically being multiparous, was associated with probable depression in the first and second trimesters as well as with having major depression in the first trimester, and in this case, it was a predictor variable. This finding may be explained by the fact that women with more children bear a greater physical and emotional burden due to the demands of caring for a larger number of family members. While some studies corroborate this association [19,23,26,32], Redinger et al. [36] found the opposite, i.e., being nulliparous was associated with gestational depression.

Having had previous miscarriages was a predictor of probable depression in the first trimester, and no relationship was found at any other time or with the presence of major depression. One possible explanation is that miscarriages often occur in the first trimester and, therefore, those women with a history of miscarriage may experience greater distress at this time because of the fear of a repeat of the situation and because, in turn, they are reminded of the previous experience. This situation could make them vulnerable to developing depressive symptoms. Once this trimester is over, these symptoms disappear. Although other authors have also found an association between previous miscarriages and antenatal depression [19,29,32], in another study [31], this relationship was not significant.

Unplanned pregnancy was associated in the second and third trimesters with both probable depression and major depression. This association has been widely documented [19,29,30,32,46]. There are several possible explanations for this. First, unplanned or unwanted pregnancies carry an enormous emotional burden [47]. Another explanation involves the socio-demographic characteristics of women who find themselves in a situation of unwanted pregnancy. As such, women may not be financially or socially prepared to cope with the demands of pregnancy [48]. These women also tend to have more unstable social environments and may feel a lack of security and support from their partner (if any) and have more marital conflict [49,50]. All of these circumstances increase the risk of antenatal depression. On the other hand, having had a negative reaction to the confirmation of pregnancy was associated with both probable depression and major depression in all three trimesters. Although this variable may be related to the previous variable (unplanned/wanted pregnancy), negative reactions do not always occur in this context. However, the explanation for this relationship may be similar, as negative reactions such as resignation and anger, among others, are an added emotional burden. Moreover, the woman may feel guilty for having such reactions, even if the pregnancy was wanted, as the idealisation of motherhood and social pressure impose that it is politically incorrect to say (or think) something negative at this time.

With regard to the presence of pregnancy complications, an association was found with both probable depression and major depression in the third trimester. Other authors [19,25] have also found this relationship. The presence of complications is a stressful life event and a psychological burden for women that favours the appearance of depressive symptomatology. It should be borne in mind that what women consider to be a complication may not be a complication from a clinical point of view. For example, some women perceive back pain or nausea as a complication, whereas for a health professional, both entities would be considered physiological or normal. Therefore, the important point in psychological terms is not what is reflected in the medical history, but the woman’s perception of the process.

Not attending maternal education classes was associated with both probable depression and major depression in the third trimester, the time of pregnancy when the women were assessed, as this is when these classes begin. Maternal education sessions aim to equip the mother and her partner with knowledge and skills to prepare them to cope with physical, emotional, and lifestyle changes in those areas of which they feel the most insecure. The mere fact of being in contact with other women in the same situation and with the same needs as well as having a healthcare professional who listens empathetically and resolves doubts can minimize the impact of the possible worries that most women have in pregnancy.

In terms of psychological variables, in all three trimesters, probable depression and major depression were both associated with a history of depression, the perceived worsening of mood in previous pregnancies, and with the presence of trait anxiety, state anxiety, and higher perceived stress.

The relationship with the presence of a history of depression has also been found by other authors in cohorts of pregnant women [23,31] as well as at specific times during pregnancy [17,26,46]. The explanation for this relationship is the existence of a vulnerability to depression that may be intensified by changes brought about by pregnancy and motherhood (e.g., sleep, rest, altered body image). Another explanation is that many women who are undergoing pharmacological treatment for a depressive episode may choose to interrupt it when they become aware of the pregnancy for fear of possible teratogenic effects on the foetus [51] and, therefore, the symptoms will possibly worsen and be prolonged throughout the perinatal period. In relation to this, in the present study, multiparous women who perceived a worsening of mood in previous pregnancies were more likely to have probable depression and major depression.

State anxiety was associated with both probable and major depression in all three trimesters of pregnancy and was also a predictor of probable depression in the first and third trimesters. This relationship is widely documented [26,33,35,36]. The main explanation is the frequent comorbidity of both disorders [35,52]. Ross et al. [53] concluded that more than 50.0% of pregnant women with depression had also been diagnosed with anxiety.

In terms of perceived stress, our data indicate that a high level of perceived stress is a predictor of both entities of depression at all points in pregnancy. This association is frequently found with other measures of stress such as the presence of stressful life events [33,36,46], and it must be borne in mind that pregnancy is considered stressful for many women because of the changes it brings about in their lives [54].

Thus, the study findings indicate that all of the psychological variables considered were associated with both probable depression and major depression at all stages of pregnancy. Therefore, it is crucial to take these variables into account in routine follow-up examinations.

Interpretation of the results obtained in the present research should take into consideration the limitation that no pre-pregnancy baseline measurements of psychological variables are available. Therefore, it is not possible to determine if there were any changes in pregnancy relative to the usual condition of the participants. The strengths of the study are mainly methodological. In particular, it is a longitudinal study (the first in Spain), which enabled us to identify both the associations between the variables and the predictors of depression status. In addition, the data were collected prospectively and individually by administering the questionnaires face-to-face to a large sample at all assessment times in order to minimize recall bias. This, in turn, resulted in low sample attrition. This is also the only study in which the prevalence and variables associated with both depressive symptomatology (assessed with a screening instrument) and major depression (assessed by clinical interview) have been analyzed together, providing a more complete view of the subject. This enabled us to understand the trajectory of two very different situations in terms of the severity of depression and to examine the similarities between the associated variables. In general, although the results do not indicate any notable differences, as the variables associated with both probable depression and major depression are similar, there are some exceptions. Specifically, previously having had a miscarriage was only associated with probable depression in the first trimester of pregnancy, while suffering pregnancy complications was only associated with major depression in the third trimester. Likewise, the level of income was only associated with probable depression in the first and second trimesters. However, lower educational level, having had a negative reaction to the confirmation of pregnancy, and all of the psychological variables were associated with both types of depression in all three trimesters. This finding reinforces the fact that the EPDS is a valid instrument for detecting women at risk of clinical depression if it is routinely implemented in pregnancy monitoring.

Regarding the clinical implications, this study enabled us to identify the variables associated with the presence of depression at different times. While some of these variables (i.e., sociodemographic variables) are unalterable, others (i.e., psychological variables) are potentially modifiable. Influencing these variables at an early stage could lead to a reduction in the prevalence and adverse consequences of depression. The study findings also enabled us to determine which variables should be assessed routinely in order to enable the necessary help to be offered at each stage, to predict which women are at the greatest risk of developing depression in pregnancy, and to develop specific interventions at the most appropriate time to prevent depression from extending to the postpartum period. The findings could thus help to improve care during the perinatal stage and prevent or reduce the likelihood of the women experiencing depression.

## 5. Conclusions

In the present study, the prevalence of probable depression in pregnancy was 23.4% in the first trimester, 17.0% in the second trimester, and 21.4% in the third trimester. The prevalence of major depression was 5.1% in the first trimester, 4.0% in the second, and 4.7% in the third trimester. The trajectories of both probable depression and major depression throughout pregnancy are therefore the same, being more prevalent in the first (in particular) and third trimesters. Regarding the variables associated with both probable depression and major depression, with the exception of variables such as having suffered previous miscarriages and pregnancy complications and/or having a lower level of income, no significant differences were found between probable and major depression. Regarding socio-demographic variables, a younger age, lower level of education, being unemployed, and the perception that pregnancy had a negative influence on employment stands out. Regarding the pregnancy-related variables, not having planned the pregnancy, negative reaction to the confirmation of the pregnancy, and not attending maternal education classes stands out. It should be noted that psychological variables were associated with probable depression and major depression in all three trimesters. In fact, the predictor variables are also mainly psychological, with perceived stress being a predictor for both conditions in all trimesters. The data indicate the importance of integrating mental health care as a part of routine pregnancy follow-up protocols. This would involve conducting assessments throughout pregnancy as early as the first trimester to detect women at risk of suffering depression.

## Figures and Tables

**Table 1:** Characteristics of women with probable depression and major depression as a function of sociodemographic variables.

	Probable Depression						Major Depression					
**First Trimester**	**No** (*n* = 436)		**Yes** (*n* = 133)				**No** (*n* = 540)		**Yes** (*n* = 29)			
	*n*	%	*n*	%	*χ* ^2^	V	*n*	%	*n*	%	*χ* ^2^	V
Age					8.825 **	0.13					0.180	
≤30	121	27.8	55	41.4			166	30.7	10	34.5		
>30	315	72.2	78	58.6			374	69.3	19	65.5		
Marital Status					1.001						4.778	
Unmarried	20	4.6	9	6.8			25	4.6	4	13.8		
Married/Cohabiting	416	95.4	124	93.2			515	95.4	25	86.2		
Level of Education					13.809 ***	0.16					4.348 *	0.09
Primary/Secondary	215	49.3	90	67.7			284	52.6	21	72.4		
University	221	50.7	43	32.3			256	47.4	8	27.6		
Employment Status					6.419 *	0.11					2.835	
Working	339	77.8	89	66.9			410	75.9	18	62.1		
Not Working	97	22.2	44	33.1			130	24.1	11	37.9		
Pregnancy Influenced Employment					14.979 ***	0.16					8.892 **	0.13
No	365	83.7	91	68.4			439	81.3	17	58.6		
Yes	71	16.3	42	31.6			101	18.7	12	41.4		
Monthly Income (Euros)					9.783 **	0.14					0.413	
≤1000	183	48.2	71	65.1			240	51.6	14	58.3		
>1000	197	51.8	38	34.9			225	48.4	10	41.7		
**Second Trimester**	**No** (*n* = 472)		**Yes** (*n* = 97)				**No** (*n* = 546)		**Yes** (*n* = 23)			
Age					8.372 **	0.12					20.727 ***	0.19
≤30	134	28.4	42	43.3			159	29.1	17	73.9		
>30	338	71.6	55	56.7			387	70.9	6	26.1		
Marital Status					0.287						0.642	
Unmarried	23	4.9	6	6.2			27	4.9	2	8.7		
Married/Cohabiting	449	95.1	91	93.8			519	95.1	21	91.3		
Level of Education					16.200 ***	0.17					8.108 **	0.12
Primary/Secondary	235	49.8	70	72.2			286	52.4	19	82.6		
University	237	50.2	27	27.8			260	47.6	4	17.4		
Employment Status					1.642						12.956 ***	0.15
Working	360	76.3	68	70.1			418	76.6	10	43.5		
Not Working	112	23.7	29	29.9			128	23.4	13	56.5		
Pregnancy Influenced Employment					24.166 ***	0.21					11.231 **	0.14
No	367	77.8	52	53.6			409	74.9	10	43.5		
Yes	105	22.2	45	46.4			137	25.1	13	56.5		
Monthly Income (euros)					7.442 **	0.12					1.872	
≤1000	199	49.1	55	65.5			243	51.4	11	68.8		
>1000	206	50.9	29	34.5			230	48.6	5	31.3		
**Third trimester**	**No** (*n* = 447)		**Yes** (*n* = 122)				**No** (*n* = 542)		**Yes** (*n* = 27)			
Age					3.335						20.637 ***	0.19
≤30	130	29.1	46	37.7			157	29.0	19	70.4		
>30	317	70.9	76	62.3			385	71.0	8	29.6		
Marital Status					0.132						0.313	
Unmarried	22	4.9	7	5.7			27	5.0	2	7.4		
Married/Cohabiting	425	95.1	115	94.3			515	95.0	25	92.6		
Level of Education					8.948 **	0.13					6.661 *	0.11
Primary/Secondary	225	50.3	80	65.6			284	52.4	21	77.8		
University	222	49.7	42	34.4			258	47.6	6	22.2		
Employment Status					3.378						11.145 **	0.14
Working	344	77.0	84	68.9			415	76.6	13	48.1		
Not Working	103	23.0	38	31.1			127	23.4	14	51.9		
Pregnancy Influenced Employment					4.066 *	0.09					0.626	
No	290	64.9	67	54.9			342	63.1	15	55.6		
Yes	157	35.1	55	45.1			200	36.9	12	44.4		
Monthly Income (euros)					1.798						0.996	
≤1000	195	50.4	59	57.8			242	51.5	12	63.2		
>1000	192	49.6	43	42.2			228	48.5	7	36.8		

* *p* < 0.05, ** *p* < 0.01, *** *p* < 0.001.

**Table 2:** Characteristics of women with probable depression and major depression as a function of pregnancy-related variables.

	Probable Depression						Major Depression					
**First Trimester**	**No** (*n* = 436)		**Yes** (*n* = 133)				**No** (*n* = 540)		**Yes** (*n* = 29)			
	*n*	%	*n*	%	*χ* ^2^	V	*n*	%	*n*	%	*χ* ^2^	V
Parity					4.928 *	0.09					10.197 **	0.13
Primiparous	270	61.9	68	51.1			329	60.9	9	31.0		
Multiparous	166	38.1	65	48.9			211	39.1	20	69.0		
Previous Abortions					11.280 **	0.14					0.024	
No	336	77.1	83	62.4			398	73.7	21	72.4		
Yes	100	22.9	50	37.6			142	26.3	8	27.6		
Pregnancy Planning					2.282						2.569	
No	56	12.8	24	18.0			73	13.5	7	24.1		
Yes	380	87.2	109	82.0			467	83.5	22	75.9		
Pregnancy Reaction					24.026 ***	0.21					13.684 ***	0.16
Positive	418	95.9	111	83.5			507	93.9	22	75.9		
Negative	18	4.1	22	16.5			33	6.1	7	24.1		
Pregnancy Complication					3.637						0.058	
No	406	93.1	117	88.0			496	91.9	27	93.1		
Yes	30	6.9	16	12.0			44	8.1	2	6.9		
**Second Trimester**	**No** (*n* = 472)		**Yes** (*n* = 97)				**No** (*n* = 546)		**Yes** (*n* = 23)			
Parity					6.959 **	0.11					1.332	
Primiparous	292	61.9	46	47.4			327	59.9	11	47.8		
Multiparous	180	38.1	51	52.6			219	40.1	12	52.2		
Previous Abortions					0.012						0.001	
No	348	73.7	71	73.2			402	73.6	17	73.9		
Yes	124	26.3	26	26.8			144	26.4	6	26.1		
Pregnancy Planning					5.575 *	0.10					8.519 **	0.12
No	59	12.5	21	21.6			72	13.2	8	34.8		
Yes	413	87.5	76	78.4			474	86.8	15	65.2		
Pregnancy Reaction					7.265 **	0.11					7.935 **	0.12
Positive	445	94.3	84	86.6			511	93.6	18	78.3		
Negative	27	5.7	13	13.4			35	6.4	5	21.7		
Pregnancy Complication					2.407						3.480	
No	400	84.7	76	78.4			460	84.2	16	69.6		
Yes	72	15.3	21	21.6			86	15.8	7	30.4		
**Third Trimester**	**No** *(n = 447)*		**Yes** (*n* = 122)				**No** (*n* = 542)		**Yes** (*n* = 27)			
Parity					1.295						0.001	
Primiparous	271	60.6	67	54.9			322	59.4	16	59.3		
Multiparous	176	39.4	55	45.1			220	40.6	11	40.7		
Previous Abortions					0.251						0.003	
No	327	73.2	92	75.4			399	73.6	20	74.1		
Yes	120	26.8	30	24.6			146	26.4	7	25.9		
Pregnancy Planning					4.048 *	0.08					5.687 *	0.10
No	56	12.5	24	19.7			72	13.3	8	29.6		
Yes	391	87.5	98	80.3			470	86.7	19	70.4		
Pregnancy Reaction					11.328 **	0.14					10.010 **	0.13
Positive	424	94.9	105	86.1			508	93.7	21	77.8		
Negative	23	5.1	17	13.9			34	6.3	6	22.2		
Pregnancy Complication					4.380 *	0.09					10.698 **	0.14
No	355	79.4	86	70.5			427	78.8	14	51.9		
Yes	92	20.6	36	29.5			115	21.2	13	48.1		
Attendance at Maternal Classes					10.357 **	0.14					6.190 *	0.10
No	149	33.3	60	49.2			193	35.6	16	59.3		
Yes	298	66.7	62	50.8			349	64.4	11	40.7		

* *p* < 0.05, ** *p* < 0.01, *** *p* < 0.001.

**Table 3:** Characteristics of women with probable depression and major depression regarding psychological variables.

	Probable Depression						Major Depression					
**First Trimester**	**No** (*n* = 436)		**Yes** (*n* = 133)				**No** (*n* = 540)		**Yes** (*n* = 29)			
	*n*	%	*n*	%	*χ* ^2^	V	*n*	%	*n*	%	*χ* ^2^	V
History of Depression					9.610 **	0.13					18.247 ***	0.19
No	412	94.5	115	86.5			506	93.7	21	72.4		
Yes	24	5.5	18	13.5			34	6.3	8	27.6		
Worsening Mood in Previous Pregnancies (*n* = 234)					4.908 *	0.15					6.049 *	0.16
No	147	87.5	50	75.8			184	86.0	13	65.0		
Yes	21	12.5	16	24.4			30	14.0	7	35.0		
Trait Anxiety					50.455 ***	0.30					10.463 ***	0.14
No	428	98.2	109	82.0			491	90.9	21	72.4		
Yes	8	1.8	24	18.0			49	9.1	8	27.6		
State Anxiety					77.467 ***	0.37					7.770 **	0.12
No	419	96.1	93	69.9			513	95.0	24	82.8		
Yes	17	3.9	40	30.1			27	5.0	5	17.2		
**Second Trimester**	**No** (*n* = 472)		**Yes** (*n* = 97)				**No** (*n* = 546)		**Yes** (*n* = 23)			
History of Depression					14.206 ***	0.16					12.267 ***	0.15
No	446	94.5	81	83.5			510	93.4	17	73.9		
Yes	26	5.5	16	16.5			36	6.6	6	26.1		
Worsening Mood in Previous Pregnancies (*n* = 234)					15.040 ***	0.25					11.106 **	0.22
No	163	89.1	34	66.7			191	86.0	6	50.0		
Yes	20	10.9	17	33.3			31	14.0	6	50.0		
Trait Anxiety					42.958 ***	0.28					38.396 ***	0.26
No	459	97.2	78	80.4			522	95.6	15	65.2		
Yes	13	2.8	19	19.6			24	4.4	8	34.8		
State Anxiety					70.950 ***	0.35					30.385 ***	0.23
No	464	98.3	75	77.3			523	95.8	16	69.6		
Yes	8	1.7	22	22.7			23	4.2	7	30.4		
**Third Trimester**	**No** (*n* = 447)		**Yes** (*n* = 122)				**No** (*n* = 542)		**Yes** (*n* = 27)			
History of Depression					12.348 ***	0.15					14.259 ***	0.16
No	423	94.6	104	85.2			507	93.5	20	74.1		
Yes	24	5.4	18	14.8			35	6.5	7	25.9		
Worsening Mood in Previous Pregnancies (*n* = 234)					5.246 *	0.16					7.619 *	0.18
No	155	87.6	42	73.7			191	85.7	6	54.5		
Yes	22	12.4	15	26.3			32	14.3	5	45.5		
Trait Anxiety					39.298 ***	0.26					41.005 ***	0.27
No	436	97.5	101	82.8			519	95.8	18	66.7		
Yes	11	2.5	21	17.2			23	4.2	9	33.3		
State Anxiety					84.056 ***	0.38					28.939 ***	0.23
No	438	98.0	90	73.8			510	94.1	18	66.7		
Yes	9	2.0	32	26.2			32	5.9	9	33.3		

* *p* < 0.05, ** *p* < 0.01, *** *p* < 0.001.

**Table 4:** Predictors of probable depression in each trimester.

**Predictors in the First Trimester**	B	WALD	*p*	OR [95% IC]
Age ≤ 30 years	0.94	4,04	0.045	2.55 [1.02–6.37]
Previous Miscarriages	1.19	7.13	0.008	3.28 [1.37–7.85]
High State Anxiety	1.38	4.70	0.03	3.97 [1.14–13,81]
High Perceived Stress	0.22	24.70	<0.001	1.24 [1.14–1.35]
Constant	−6.49	38.88	<0.001	0.002
Cox and Snell R2	0.343			
Nagelkerke’s R2	0.501			
**Predictors in the Second Trimester**				
Probable Depression in the 1st Trimester	2.61	21.41	<0.001	13.61 [4.51–41.14]
Higher Perceived Stress in 1st Trimester	0.12	6.53	0.011	1.14 [1.03–1.24]
Higher Perceived Stress in 2nd Trimester	0.30	26.46	<0.001	1.35 [1.21–1.52]
Constant	−5.90	29.98	<0.001	0.003
Cox and Snell R2	0.402			
Nagelkerke’s R2	0.611			
**Predictors in the Third Trimester**				
Major Depression in the 1st Trimester	1.69	6.49	0.011	5.43 [1.48–19.95]
Probable Depression in the 2nd Trimester	1.82	16.47	<0.001	6.19 [2.47–14.91]
High State Anxiety in 3rd Trimester	1.59	4.49	0.034	4.88 [1.13–21.13]
Higher Perceived Stress in 3rd Trimester	0.15	15.57	<0.001	1.16 [1.08–1.25]
Constant	−5.22	36.82	<0.001	0.005
Cox and Snell R2	0.368			
Nagelkerke’s R2	0.555			

**Table 5:** Predictors of major depression in each trimester.

**Predictors in the First Trimester**	B	WALD	*p*	OR [95% IC]
Negative influence of Pregnancy on Work Situation	1.16	5.31	0.021	3.20 [1.19–8.63]
High Perceived Stress	0.12	12.21	<0.001	1.13 [1.05–1.21]
Constant	−5.45	37.28	<0.001	0.004
Cox and Snell R2	0.094			
Nagelkerke’s R2	0.210			
**Predictors in the Second Trimester**				
Age ≤ 30 years	1.71	5.33	<0.001	1.26 [1.13–23.42]
High Perceived Stress in the 2nd Trimester	0.24	15.66	<0.001	1.27 [1.13–1.42]
Constant	−9.13	27.10	<0.001	0.000
Cox and Snell R2	0.127			
Nagelkerke’s R2	0.398			
**Predictors in the Third Trimester**				
Age ≤ 30 years	1.98	4.98	0.026	7.23 [1.27–41.08]
High Perceived Stress in the 2nd Trimester	0.15	4.22	0.04	1.16 [1.01–1.34]
High Perceived Stress in 3rd Trimester	0.16	4.20	0.04	1.18 [1.01–1.37]
Constant	−11.51	20.60	<0.001	0.000
Cox and Snell R2	0.147			
Nagelkerke’s R2	0.485			

## Data Availability

The data presented in the current study are available from the authors upon request.
